# The Neuropsychological Profile of Attention Deficits of Patients with Obstructive Sleep Apnea: An Update on the Daytime Attentional Impairment

**DOI:** 10.3390/brainsci10060325

**Published:** 2020-05-27

**Authors:** Paola Angelelli, Luigi Macchitella, Domenico Maurizio Toraldo, Elena Abbate, Chiara Valeria Marinelli, Michele Arigliani, Michele De Benedetto

**Affiliations:** 1 Lab of Applied Psychology and Intervention, Department of History, Society and Human Studies, University of Salento, 73100 Lecce, Italy; macchitella.luigi@libero.it (L.M.); elena.abbate2@gmail.com (E.A.); chiaravaleria.marinelli@unisalento.it (C.V.M.); 2 “V. Fazzi” Hospital Rehabilitation Department, Respiratory Care Unit, ASL, 73100 Lecce, Italy; d.torald@tin.it; 3 “V. Fazzi” Hospital, ENT, ASL, 73100 Lecce, Italy; michele.mariano.arigliani@gmail.com (M.A.); micheledebenedetto@hotmail.it (M.D.B.)

**Keywords:** obstructive sleep apnea (OSA), chronic intermittent hypoxia (CIH), excessive daytime sleepiness (EDS), alertness, vigilance, selective attention, divided attention

## Abstract

Introduction: Patients with obstructive sleep apnea (OSA) suffer from several neurocognitive disturbances. One of the neuropsychological processes most investigated in OSA patients is attention, but the results have been controversial. Here, we update the attention profile of OSA patients with the final aim to improve attention assessment, with a possible impact on clinical and medical-legal practices, in terms of which attention subdomains and parameters need consideration and which one is a high-risk OSA phenotype for attention dysfunctions. Method: For this purpose, we assessed 32 previously untreated OSA patients (26 men and 6 women) under 65 years of age (mean age 53.2 ± 7.3; mean education level 10.4 ± 3.4 years) suffering from moderate to severe sleep apnea and hypopnea (mean apnea-hypopnea index (AHI) 45.3 ± 22.9, range 16.1–69.6). A control group of 34 healthy participants matched with OSA patients for age, education level, and general cognitive functioning were also enrolled. The OSA patients and healthy participants were tested through an extensive computerized battery (Test of Attentional Performance, TAP) that evaluated intensive (i.e., alertness and vigilance) and selective (i.e., divided and selective) dimensions of attention and returned different outcome parameters (i.e., reaction time, stability of performance, and various types of errors). Data analysis: The data were analyzed by ANCOVA which compared the speed and accuracy performance of the OSA and control participants (cognitive reserve was treated as a covariate). The possible mechanisms underlying attention deficits in OSA patients were examined through correlation analysis among AHI, oxygenation parameters, sleepiness scores, and TAP outcomes and by comparing the following three phenotypes of patients: severe OSA and severe nocturnal desaturators (AHI^++^D^+^), severe OSA nondesaturators (AHI^++^D^−^), and moderate OSA nondesaturators (AHI^+^D^−^). Results: The results suggest that the OSA patients manifest deficits in both intensive and selective attention processes and that reaction time (RT) alone is ineffective for detecting and characterizing their problems, for which error analysis and stability of performance also have to be considered. Patients with severe OSA and severe hypoxemia underperformed on alertness and vigilance attention subtests. Conclusions: The data suggest the importance of evaluating attention deficits among OSA patients through several parameters (including performance instability). Moreover, the data suggest a multifaceted mechanism underlying attention dysfunction in OSA patients.

## 1. Introduction

Although obstructive sleep apnea (OSA) is one of the most frequent sleep breathing disorders, it is still largely unknown and underdiagnosed (e.g., [1]). It is characterized by repeated complete or partial collapse of the upper airway during sleep that causes episodes of apnea (cessation of breathing for 10 s or longer) or hypopnea (significant breathing reduction), oxygen desaturation, and repetitive microarousals. A recent worldwide epidemiological study (carried out in 16 countries) indicated that 936 million people aged 30–69 years suffer from severe OSA and 425 million people are affected by moderate to severe OSA [2]. OSA is a risk factor for cardiac and cerebrovascular diseases [3], as well as motor vehicle accidents, low work performance and occupational accidents (e.g., [4,5,6,7]). Moreover, neuropsychological and neuroimaging studies have demonstrated that OSA is associated with impairment of several cognitive functions (including attention, memory, and executive functions; for meta-analysis see [8,9,10,11]) as well as the brain structures underlying these functions (e.g., hippocampus, thalamus, prefrontal cortex, cingulate gyrus, and frontoparietal regions; e.g., [12,13,14,15,16]). Attention is among the neuropsychological processes most investigated in OSA patients, since it pervades the entire information-processing activities directing attention resources towards a target (selectively) and guaranteeing a quantity of resources adequate for the complexity of the task (intensity). Several meta-analyses have shown that OSA patients are affected by attention deficits (see, e.g., [8,10]). However, even if there is evidence of cognitive and attention impairments in patients with sleep breathing disorders (including OSA), there are remarkable differences between various subfunctions within each cognitive domain [9]. In fact, results regarding the efficiency of the different attention processes in OSA vary such as vigilance performance was found to be defective in some but not all studies and small to moderate deficits were found in focused and sustained attention, while divided attention did not seem impaired [9]. However, there are also studies (e.g., [14]) that failed to find any attention deficits in patients suffering from moderate to severe OSA.

The inconsistent results among studies could be due to numerous factors, including differences in the tasks used to assess the same attention process (see, for example, [14]), different comorbidities (e.g., hypertension), severity of disease, age, and compensatory mechanisms such as cognitive reserve (see, e.g., [17,18,19]). All of these factors can affect patient performance. In particular, cognitive reserve is the ability to optimize and maximize performance through the following two mechanisms: recruitment of brain network and compensation by alternative cognitive strategies (see, e.g., [20,21,22,23]). Thus, Yaouhi et al. [14] explained the contrasting results between an evident metabolic and structural brain alteration and the absence of attention impairment in terms of subjects’ cognitive reserve, which could have acted as a protective factor. Unfortunately, cognitive reserve has rarely been considered and controlled. In the study by Yaouhi et al., cognitive reserve was not assessed, although it was postulated as a possible mediating factor. To our knowledge, only one study has comprehensively evaluated patients’ cognitive reserve [24], while in others it was improperly measured by intelligence [18,25].

Another important issue concerns the outcome parameters (reaction time, stability of performance, and response accuracy) examined in the various studies, which can differ in their degree of sensitivity for revealing the attention impairment. On the one hand, simple reaction times (RTs) in sustained attention tasks were found to be reduced in some (e.g., [26]) but not all studies [9,27,28]. On the other hand, some data underline that deficits in sustained attention and/or vigilance are revealed only by measuring performance accuracy or performance instability rather than reaction time [9,29,30,31,32]. Therefore, it appears that RT analysis alone does not fully capture vigilance or sustained attention deficits. On the contrary, errors have been proven to be more reliable as valid indices in the assessment of diurnal attention impairment [31], as they are important indicators of inattention (e.g., omissions) and impaired selectivity of attention or reduced control of response (e.g., false responses). Interestingly, in sustained attention performance, one important aspect of performance change is an increase of “lapses” (i.e., reaction times greater than twice the subject’s baseline mean), even though subjects should be capable of normal timely and accurate responses [33]. Following the “state instability” hypothesis, originally formulated to study performance change in neurobehavioral tasks due to sleep deprivation, increased variability is due to the influence of sleep initiating mechanisms on the endogenous capacity to maintain attention and alertness, thereby creating an unstable state that fluctuates within seconds and cannot be characterized as either fully awake or asleep. However, response variability, which needs performance to be sampled very frequently, has been largely ignored in OSA research in favor of global measures of performance (e.g., speed or accuracy). To our knowledge, only a few studies have taken into account performance instability in vigilance and sustained attention tasks and found that patients’ RTs became unstable with time (e.g., [26,27]), suggesting a fatigue state. In conclusion, it seems that there is increased interest in disclosing the most sensitive indices to detect attention deficits in OSA patients and to clarify which of the components of a task (e.g., motor or decisional stage) could explain the performance (e.g., [24,26,34].

In addition, the mechanisms underlying cognitive deficits in OSA patients are still being debated. Repetitive episodes of apnea and hypopnea determine both chronic intermittent hypoxia (CIH) and sleep fragmentation induced by frequent arousals. CIH is an important physiological mechanism that could link OSA to vascular, cerebral, and neurocognitive deficits [35,36,37,38,39,40]. In particular, oxidative stress induced by CIH and increased blood clotting (caused by changes in the rheological properties of blood and plasma) are important physiological mechanisms of the disease and cause cerebrovascular complications and impairments in several brain regions [37,38,39,41,42,43]. Thus, hypoxia is considered to be the main factor underlying neurocognitive deficits in OSA (for a recent meta-review, see [40]. Furthermore, frequent nocturnal sleep apneas and hypopneas also result in sleep fragmentation and altered sleep architecture, which are believed to contribute to the prominent symptom of excessive daytime sleepiness (EDS) (The American Academy of Sleep Medicine (AASM, [44]) defines excessive daytime sleepiness (EDS) as the inability to maintain wakefulness and alertness during the major) [45], which, in turn, predicts some cognitive deficits [27,46,47,48]. However, the mechanisms underlying EDS are unclear [45,49,50,51]. EDS could be due to both sleep fragmentation and brain injuries induced by CIH ([41,42,45,50,52,53,54,55]; for a recent review, see [51,56]). In any case, several authors have stressed that EDS in OSA could be involved in only some cognitive processes, such as vigilance and alertness (see, e.g., [46,57,58]). More recently, Shpirer et al. [36] demonstrated that hypoxemia, but not EDS, is correlated with attention dysfunction in OSA patients; performance speed and accuracy on a sustained and selective attention test significantly correlated with the number of apneas and hypopneas (AHI) and other parameters of nocturnal hypoxemia. Moreover, it was found that patients with significant hypoxemia underperformed on attention tests, while patients with and without sleepiness did not differ. Thus, it appears that hypoxemia could be involved not only in executive dysfunction but also in sustained attention deficit ([36]; see also [59]), and parameters of oxygenation could have similar or greater usefulness than the AHI in determining cognitive dysfunctions. Labarca et al. [60] also proposed that parameters of oxygenation should be used to describe a high-risk phenotype of OSA. Currently, various parameters of nocturnal hypoxemia have been found to be informative as follows: cumulative sleep time percentage with oxygen saturation <90% (T90; e.g., [36,60]); occurrence of desaturation events per hour (oxygen desaturation index (ODI); e.g., [28,56,61]; and lowest values of oxygen saturation during a sleep study (e.g., [60,62]).

However, it has also been found that hypoxemia and sleep fragmentation did not predict neuropsychological deficits in OSA, and it was suggested that this lack of a relationship could be explained by several interindividual factors (e.g., age, premorbid intelligence, comorbidities such as obesity and cardiovascular disease) as well as low sensitivity of routine indices assessing hypoxia and sleep fragmentation ([18,19] and references therein).

In the present study, we used a battery of attention tests to objectively characterize the different dimensions of attention and analyzed both speed and accuracy performance. Moreover, we controlled for some important variables such as age, OSA severity, presence of cognitive decline, relevant comorbid pathologies, and cognitive reserve. Finally, we tried to clarify the relationship between the various attention processes and several important clinical dimensions of OSA pathology. The final aim was to improve the attention assessment of OSA patients, with a possible impact on clinical and medical-legal practices, in terms of which attention subdomains and parameters need consideration and which one is a high-risk OSA phenotype for attention dysfunctions.

## 2. Methods

### 2.1. Participants

The participants were 32 previously untreated OSA patients (26 men and 6 women, with a mean age of 53.2 ± 7.3 years and mean education level of 10.4 ± 3.4 years) consecutively admitted to the Department of Otorhinolaryngology and the Respiratory Rehabilitation Care Unit of “V. Fazzi” Hospital Lecce (Italy) from October 2017 to November 2018. All patients had received a diagnosis of OSA in accordance with the International Classification of Sleep Disorders which was verified with an overnight polygraphic recording evaluation [1].

All patients underwent a clinical interview about their medical history, and their medical records (charts) were carefully examined. Patients were excluded from the sample for the following reasons: (i) they were in current treatment with continuous positive airway pressure (CPAP); (ii) they had a significant medical condition (e.g., diabetes mellitus, heart disease, tumor) or other psychiatric, neurological or sleep disorder (depression, ictus, epilepsy); (iii) they were taking medications that could adversely affect cognitive function (e.g., benzodiazepines or antidepressants); (iv) they had below-normal performance on the Mini-Mental State Examination (according to [63]); or (v) they were over 65 years of age. As this study concerned moderate and severe sleep apnea, only patients with an AHI ≥ 15 were included. Daytime sleepiness was measured by the Epworth Sleepiness Scale (ESS; [64,65]), which is the most widely used questionnaire that provides an estimation of subjective daytime sleepiness and concentration disorders as a consequence of OSA. There is no uniform system for interpreting the ESS, but a score >10 indicates significant EDS and >15 serious sleepiness [66]. Body mass index (BMI) was calculated as the ratio of body weight/body height (in kilograms per square meter). Obesity was diagnosed at BMI ≥ 29.9 kg/m^2^.

Cognitive reserve was assessed through the Cognitive Reserve Index questionnaire (CRIq; [21]). The CRIq includes demographic data and 20 items grouped into 3 sections (education, occupation, and leisure time), and provides a standardized measure of cognitive reserve accumulated by individuals throughout their lives.

A healthy control group was also enrolled. Control participants included 34 volunteers who were matched with OSA patients for age (50.3 years, SD 6.14), education level (11.7 years, SD 2.7), cognitive reserve index, and Mini-Mental State Examination score [63]. Ear, nose, and throat (ENT) investigation in the control group did not highlight otorhinolaryngological diseases. Furthermore, control participants did not present any history of snoring or sleep complaints or symptoms or combinations of symptoms of OSA on a 5-point questionnaire investigating habitual snoring, morning fatigue, hypertension, neck size in centimeters (x = 16.64, SD = 0.5, range 15.5–17.5) and body mass index (x = 25.8, SD = 2.1, range 22.4–32). The questionnaire was a modified version of the known STOP-Bang questionnaire [67]. There were no participants who complained of daytime sleepiness at the ESS.

This study was approved by the Ethical Committee of “Vito Fazzi” Hospital, Lecce (verbal No. 39, 28 July 2016). All subjects gave their written informed consent in accordance with the Declaration of Helsinki.

### 2.2. Assessment of Patients with Sleep-Disordered Breathing

OSA was confirmed by recorded polygraphic evaluation with an apnea-hypopnea index (AHI) of >5 apneas/hour of sleep according to the diagnostic criteria of the American Academy of Sleep Medicine [68]. The definitions of apnea and hypopnea were based on standard criteria [1]. The AHI scores per hour of sleep were indicative of mild (5 ≥ AHI < 15), moderate (15 ≥ AHI < 30) or severe (AHI ≥ 30) OSA according to Berry et al. [1]. Portable monitoring (PM) was used as an alternative to polysomnography for OSA diagnosis [69,70]. For appropriately selected patients, evidence has been accumulating that PM is a reasonable substitute for in-laboratory polysomnography. Indices of oxygen saturation, snoring, air flow, thoracic and abdominal respiratory movements, heart rate including ECG in real-time mode, and body position were assessed polygraphically (Embletta PDS recording system, Broomfield, CO, USA). Each recording was performed between 23:00 and 06:00. The signals, which were saved in a digital recorder, were computer analyzed and validated by the physician the morning after the recording. In addition, the following parameters of nocturnal arterial oxygen saturation were computed: mean percentage of oxygen saturation (mean SaO_2_), oxygen desaturation index (ODI, number of oxyhemoglobin desaturations >4% per hour of sleep), cumulative sleep time percentage spent with SaO_2_ < 90% (T90), and lowest value of oxygen saturation (nadir SaO_2_ (NSaO_2_) or lowest SaO_2_). Patients showing T90 ≥ 30% and NSaO_2_ ≤ 85% were defined as desaturators (D^+^), and other patients as nondesaturators (D^−^) (e.g., [60,71], for similar criteria).

### 2.3. Attention Assessment

We assessed both intensive attention processes, such as alertness and vigilance, and selective attention processes, such as selective and divided attention.

Alertness refers to the condition of general wakefulness that enables a person to respond quickly and appropriately to a sudden request for action. Intrinsic (also called endogenous) alertness refers to the cognitive (top-down) control of arousal; it is typically assessed by simple reaction time to a visual or auditory stimulus without a warning signal. By contrast, phasic (also called exogenous) alertness is the ability to increase one’s general level of attention for a short period in response to a cue or warning signal preceding the target stimulus [72,73]. Vigilance involves maintaining a certain level of arousal and alertness during a long task in order to detect infrequent but relevant stimuli (such as those that occur when driving a car on a highway at night (see [73,74,75,76]). Finally, selective attention allows enhanced processing of attended or salient stimuli/features; at the same time, it inhibits the treating of irrelevant information [77,78], and the ability to perform two tasks simultaneously, in the same or different sensory modalities, is defined as divided attention [76,79].

The attention subfunctions were assessed by 4 subtests from the Test of Attentional Performance (TAP; [80]). All tests are computerized and include various parameters that allow evaluation of performance speed and accuracy. Performance speed is assessed by reaction time variables (median, mean) that are calculated only for valid responses. Reaction time provides information about the general speed of processing and possible processing attenuation; the SD of RT is a measure of the stability or instability of the level of performance. In general, it is caused by strong variation in reaction times or isolated “lapses of attention.” Performance accuracy is evaluated by the number of valid responses and various types of errors depending on the attention test. Errors can help characterize attention difficulties; in fact, omissions (lack of responses to target stimuli) are an important indicator of inattention, false responses (responses to non-target stimuli) can indicate impaired selectivity of attention or reduced control of response, and anticipation (responding in less than 100 ms) can indicate an inability to inhibit impulsive and delayed reactions (in excess of the normal area as defined by the individual mean), and all are measures of lapses of attention.

The following subsections provide descriptions of the four subtests.

#### 2.3.1. Alertness

This test measures reaction time (RT) to a simple visual target with or without a warning signal (tone). A cross appears in the middle of the computer screen and the subject has to press a button as rapidly as possible. The order of block presentation is ABBA: A is the block without a warning signal and B is the block with warning signal. A total of 80 trials were presented to each participant. The test lasted 4.5 min. Regarding errors, only anticipations, omissions, and delayed responses were furnished.

A test-specific parameter, the index of phasic alertness, is computed by comparing reaction times with warning and without warning. This parameter reports the increase in the level of attention when it is greater than null.

The main parameters for this test are median RTs of the subtests with and without warning; SDs of RTs; and number of valid responses, anticipations, and delayed responses.

#### 2.3.2. Vigilance

At the center of the screen, a horizontal bar, 3 cm long and 0.3 cm wide, moves regularly up and down with a 1.8 cm oscillation. The subject had to press the button when the bar showed a larger oscillation (~3.5 cm). The target rate was about one stimulus per minute for a total of 36 targets. The vigilance test lasted 30 min. Separate data were available for the entire test period and for each 5 min period, thus permitting evaluation of how the level of performance changes as a function of time.

The main parameters for this test are median RT; SD of RTs; and number of valid responses, omissions, and false reactions for each 5 min period and the whole test.

#### 2.3.3. Go/No Go

**Figure 1:**
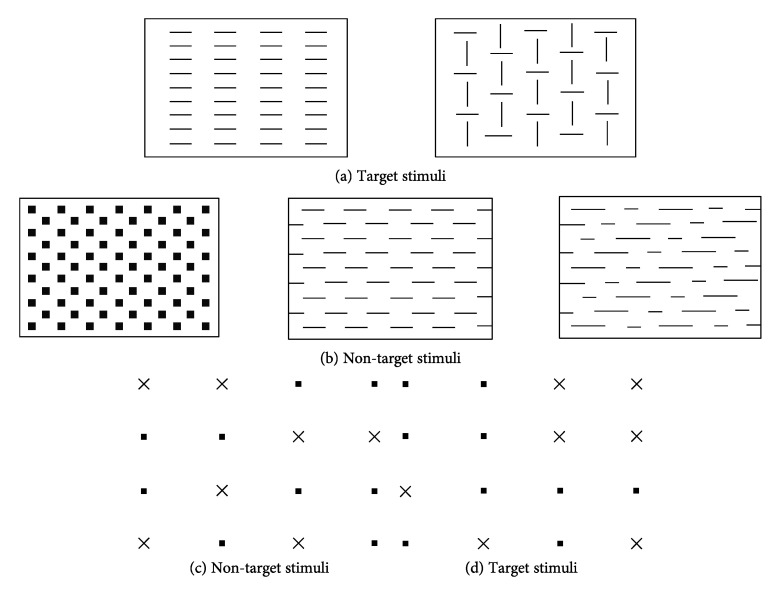
(**a**,**b**): Go/no go stimuli; (**c**,**d**): Non-target and target stimuli of divided attention test.

One 3 × 3 cm square appears in the middle of the screen. There are 2 target stimuli (see Figure 1a,b). The subject had to press the button when a target was presented and not press the button when a non-target was presented. A total of 60 trials were presented; 24 were critical. The test lasted 2.45 min.

The main parameters for this test are median RT; SD of RTs; and number of valid responses, omissions, and false reactions.

#### 2.3.4. Divided Attention

Two tasks, one visual and one auditory, were presented simultaneously. In the visual task, a matrix of 16 dots (4 × 4) with 7 little x’s were displayed on the screen (see Figure 1c,d). The subject had to press a key when 4 x’s form a square. In the auditory task, a series of 2 sounds, one high and one low, was presented (Di-Da-Di-Da, etc.); the task was to detect a variation in the sequence (Di-Di or Da-Da). RTs and number of omissions were the measures considered. A total of 300 stimuli were presented (100 visual and 200 auditory); of these, 33 were crucial (17 visual and 16 auditory). The test lasted 3.25 min.

The main parameters for this test are median RT; SDs of RTs; and number of valid responses, false reactions, omissions, and delayed reactions.

### 2.4. Procedure

Participants were tested individually in the morning in a quiet room. Stimuli were presented on the screen of a personal computer (PC) about 60 cm away from the patient. Participants responded by pressing a button connected to the PC. This allowed measurement of RTs and accuracy. Instructions for each test were given aloud and a short sequence of practice trials preceded each test. Brief pauses were allowed between tests. The various attention tasks were administered in random order to minimize feelings of fatigue and discouragement.

### 2.5. Data Analysis

Descriptive statistics were performed for relevant demographic and clinical variables of OSA patients and healthy subjects, and group means were compared by ANOVA.

For the TAP battery, the median RT of the alertness, go/no go, divided attention, and vigilance tests were corrected for age and education following the manual (for details, see [80]. Separate ANOVAs were used to compare RTs (corrected median RTs); SDs of RTs; valid responses; and errors in the alertness, vigilance, go/no go and divided tests. In these ANOVAs, group was treated as a between factor (OSA patients vs. healthy controls), the outcome parameters (RTs, SDs of RTs, valid responses, and errors) as dependent variables, and cognitive reserve as covariate. When present, the effects of the conditions/tasks (for the alertness and divided attention tests) and intervals (for the vigilance test) were evaluated in OSA patients and healthy controls as repeated measures as follows: (1) the effect of warning in the alertness test (two levels: warning vs. no warning); (2) the effect of the task in the divided attention test (two levels: auditory vs. visual); and (3) the effect of the time course in the vigilance test (6 levels: 0–5, 5–10, 10–15, 15–20, 20–25, and 25–30 min).

All previously described analyses were replicated, with the CRI parameter added as covariate in ANCOVA, in order to examine the attention profiles of the 2 groups when cognitive reserve was partially excluded.

In both ANOVA and ANCOVA, interactions were explored with the post-hoc Tukey test. Additionally, the post-hoc power analysis was performed, with an asterisk placed on F values that exceeded the critical value, given the probability of a Type I error set at 0.05 and a Type II error set at 0.80.

In order to better understand the relationship between the attention performance and severity of OSA, we carried out 2 analyses: Pearson correlation between AHI, nocturnal parameters of hypoxemia, ESS scores, and TAP data; and univariate ANOVA with 3 subgroups of patients classified according to severity of AHI (moderate vs. severe) and oxygen desaturation (desaturator vs. nondesaturator; see Section 2.2). In particular, after inspecting individual pieces of data, patients were grouped as severe OSA desaturators (AHI^++^D^+^), severe OSA nondesaturators (AHI^++^D^−^), or moderate OSA nondesaturators (AHI^+^D^−^). Descriptive statistics were performed for relevant demographic, and clinical variables for the 3 subgroups of patients, and means were compared by univariate ANOVA. The attention profile was studied, replicating the repeated measures ANOVA, with subgroup (OSA phenotype) as the between factor. Due to the small number of patients in each group, the analyses were not replicated with cognitive reserve as covariate. Interactions were explored with the post-hoc Tukey test. The post-hoc power analysis was performed; an asterisk was placed on F values that exceeded the critical value, given the probability of a Type I error set at 0.05 and a Type II error set at 0.80.

## 3. Results

### 3.1. Demographic and Clinical Characteristics

Table 1 reports the main demographic and clinical data of OSA patients and healthy controls. The two groups were comparable for all variables considered.

Table 2 reports the means of patients’ AHI and the parameters of hypoxemia. The mean ESS for OSA was 12.5 ± 2.8 (range 5–20) and for controls was 2.2 ± 0.9 (range 1–4), with the difference being significant (F_(1,64)_ = 39.72, *p* < 0.0001). A total of 26 OSA patients (81%) had ESS scores ≥ 10.

Patients had a mean BMI of 34.6 ± 7.9 (range 22.3–69.6) and 23 patients (78%) had values above 30 (the cut-off for obesity). The OSA patients and controls (mean 25.8, range 22.4–32) differed significantly in BMI (F_(1,64)_ = 37.33, *p* < 0.0001).

Table 3 reports the Pearson correlations between AHI, nocturnal parameters of hypoxemia, and ESS scores of OSA patients. AHI correlated significantly with all nocturnal variables of hypoxemia, whereas ESS scores did not correlate with any index.

Inspection of individual parameters showed that 23 patients (72%) had severe OSA (AHI ≥ 30) and nine (28%) had moderate OSA (15 ≥ AHI < 30). Interestingly, ESS scores of patients with moderate (12.11 ± 1.6) and severe (12.85 ± 3.4) AHI did not differ (F < 1). The parameters of nocturnal hypoxemia showed that 24 patients (75%) had an ODI ≥ 30 (five patients ≥ 15 ODI < 30 and two patients ≥ 5 ODI < 15). Moreover, 13 patients (41%) had T90 values ≥30% and 25 patients (78%) had NSaO_2_ ≤ 5%. Taking T90 and NSaO_2_ values together, a total of 13 patients (41%) could be defined as desaturators (T90 ≥ 30% and NSaO_2_ ≤ 85%) and 21 patients (66%) as nondesaturators. See Table 4 for a cross-tabulation of patients as a function of moderate vs. severe AHI and hypoxemia severity. One patient with moderate OSA was classified as a desaturator and the other patients were grouped into the following three subgroups: 10 AHI^++^D^+^, 13 AHI^++^D, and 8 AHI^+^D^−^. Comparisons of the main demographic and clinical variables (see Table 5) revealed that the three groups were comparable for age, education, Mini-Mental State Examination score, cognitive reserve, and BMI. Interestingly, the mean ESS scores did not differentiate patients with different OSA severity and degrees of desaturation. AHI^++^D^+^ presented higher AHI and ODI than AHI^++^D^−^ (at least *p* < 0.01) and AHI^+^D^−^ (at least *p* < 0.0001). AHI^++^D^−^ also presented higher AHI and ODI than AHI^+^D^−^ (*p* < 0.0001). The mean SaO_2_ was lower in AHI^++^D^+ a^ as compared with AHI^++^D^−^ and AHI^+^D^−^ (at least *p* < 0.01), with the latter two groups being comparable. As mentioned, AHI^++^D^+^ had higher T90 and lower NSaO_2_ with respect to AHI^++^D^−^ and AHI^+^D^−^ (at least *p* < 0.01), with the latter two groups being comparable.

### 3.2. Alertness

In both the RT and SD analyses, the repeated measures ANOVA (OSA vs. healthy controls) highlighted the main effect of group for RTs (274.2 vs. 243.5 ms, F_(1,64)_ = 5.67*, *p* < 0.05) and SDs of RTs (47.8 vs. 34.7 ms, F_(1,64)_ = 6.80*, *p* < 0.01), indicating that OSA patients had longer RTs and greater performance instability than the controls. A main effect of warning was present in RTs (F_(1,64)_ = 7.84*, *p* < 0.01), but not SDs (F_(1,64)_ = 1.20, n.s.), with shorter RTs in the warning than the no-warning condition (241 vs. 250 ms, F_(1,64)_ = 7.84*, *p* < 0.01) in both OSA and control participants (group-by-warning interaction was not significant at F < 1). Likewise, the index of phasic alertness was the same for both groups (0.04).

Concerning accuracy, the effect of group and its interaction was not significant for the number of correct responses (F_(1,64)_ = 0.79, n.s.), anticipations and delayed responses (all F < 1). The accuracy of OSA patients, with longer response times and greater fluctuations, was similar to that of the control participants.

When analyses were replicated with the cognitive reserve index as covariate, all results were replicated, except for the main effect of warning on RTs, which was still not significant (F_(1,62)_ = 0.29, n.s.); in all analyses the covariate was not significant (F < 1).

**Figure 2:**
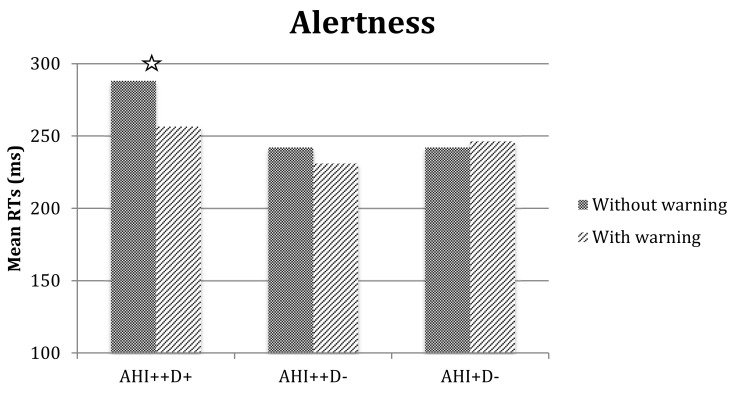
Alertness reaction times in no-warning and warning conditions for three groups of OSA patients. Legend: Stars indicate significant differences.

The repeated measures ANOVA with three subgroups of patients (AHI^++^D^+^, AHI^++^D^−^, and AHI^+^D^−^), confirmed the main effect of warning (with shorter RTs in the warning than the no-warning condition, 244.75 ms vs. 257.57 ms, respectively, F_(1,28)_ = 13.69*, *p* < 0.001), but also the group-by-warning interaction was significant (F_(2,28)_ = 8.31*, *p* < 0.01). Exploration of the interaction (see Figure 2) revealed that the AHI^++^D^+^ subgroup had higher RTs in the no-warning with respect to the warning condition (*p* < 0.01), but their speed performance increased in the warning condition and became comparable to that of other groups. There were no effects or interactions that reached significance in the analysis of SDs, valid responses, anticipations, or delayed responses.

### 3.3. Vigilance

ANOVA of the 30 min period of the vigilance test (Table 6a) revealed no differences in RTs and SDs of RTs between groups. However, the OSA patients had difficulty adapting their reaction times to the task; they presented a clear speed–accuracy trade-off, preferring rapidity at the expense of accuracy. In fact, they made fewer valid responses (31.6 vs. 33.8, F_(1,64)_ = 4.49*, *p* < 0.05) and had an increased rate of omissions (3.62 vs. 1.2, F_(1,64)_ = 7.30*, *p* < 0.01). The number of false responses was comparable in both groups (F < 1).

Repeated measures ANOVA of RTs showed a main effect of interval (F_(5,300)_ = 7.10*, *p* < 0.0001); participants had slightly longer RTs in the 0–5 min interval with respect to the other intervals (at least *p* < 0.001), which did not differ. The effects of group (F_(1,64)_ = 1.19, n.s.) and group-by-interval (F < 1) were not significant.

**Figure 3:**
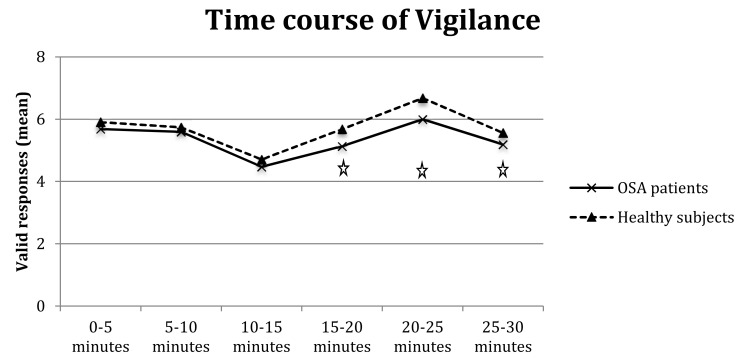
Valid responses of OSA patients and healthy controls in six vigilance intervals. Legend: Stars indicate significant differences.

The analysis of valid responses showed a main effect of group (F_(1,64)_ = 1.38, *p* < 0.05) and interval (F_(5,320)_ = 5.38*, *p* < 0.0001) and a significant group-by-interval interaction (F_(5,320)_ = 2.24, *p* < 0.05). The interaction (Figure 3) highlighted that OSA patients had fewer valid responses than controls in medium/long intervals, particularly 15–20 min (F_(1,64)_ = 6.40*, *p* < 0.01) and 20–25 min (F_(1,64)_ = 6.23*, *p* < 0.01), with a tendency towards significance also for 25–30 min (F_(1,64)_ = 3.43, *p* = 0.07).

**Figure 4:**
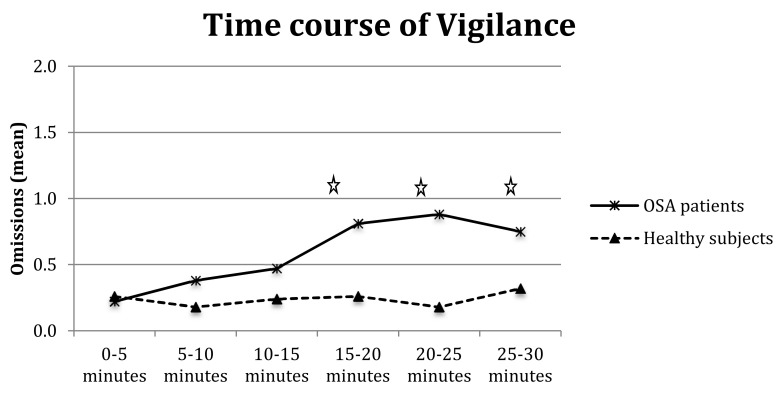
Omissions of OSA patients and healthy controls in six vigilance intervals. Legend: Stars indicate significant differences.

Furthermore, the analysis of omissions showed the significance of the main effects of group (F_(1,64)_ = 5.60*, *p* < 0.05), interval (F_(5,320)_ = 2.82*, *p* < 0.01), and group-by-interval interaction (F_(5,320)_ = 2.64*, *p* < 0.05); as shown in Figure 4, OSA patients made significantly more omissions than controls at longer intervals, particularly 15–20 min (F_(1,64)_ = 5.14*, *p* < 0.05), 20–25 min (F_(1,64)_ = 9.12* *p* < 0.01), and 25–30 min (F_(1,64)_ = 4.63*, *p* = 0.05).

There were no effects or interactions that reached significance in the analysis of false responses (all F < 1).

When analyses were replicated with the cognitive reserve index as covariate, all results were replicated, and the covariate was not significant (F < 1).

**Figure 5:**
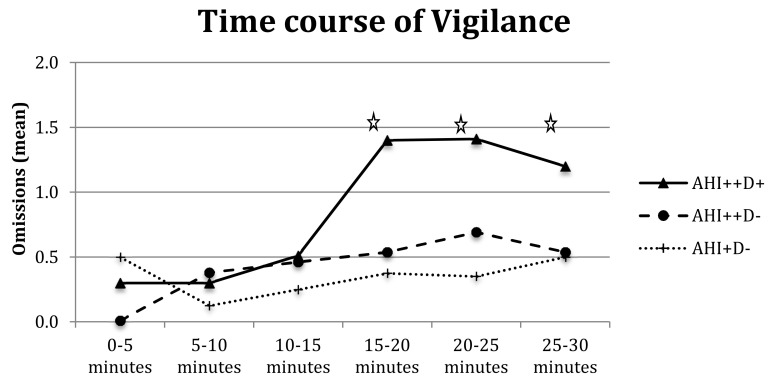
Omissions in six intervals of vigilance test for three groups of OSA patients.

The repeated measures ANOVA on RTs with three subgroups of patients (AHI^++^D^+^, AHI^++^D^−^, and AHI^+^D^−^) showed a main effect of group (F_(1,28)_ = 3.67, *p* < 0.05) with AHI^++^D^+^ presenting higher RTs than AHI^++^D^−^ (505.30 vs. 401.57 ms, *p* < 0.05) and the other two groups being comparable (401.57 vs. 443.20 ms, respectively). The analysis confirmed a main effect of interval for RTs (with the 0–5 min RTs slightly slower than the 5–10 min, F_(5,28)_ = 3.77*, *p* < 0.01), valid responses (F_(5,28)_ = 18.3*, *p* < 0.001) and omissions (F_(5,28)_ = 3.93*, *p* < 0.01), with a decrease in valid responses in the medium and long intervals (10–15, 15–20, and 25–30 min, at least *p* < 0.05) and a significant increase of omissions from 15–20 min onwards (F_(5,28)_ = 3.77*, *p* < 0.01). Group-by-interval interaction was not significant for RTs and valid responses (both F < 1) but tended towards omissions (F_(5,28)_ = 1.77, *p* = 0.07, Figure 5), with AHI^++^D^+^ committing more omissions during medium and long intervals (15 min on words).

There were no main effects or interactions that reached significance in the analysis of SDs and false reactions.

### 3.4. Go/No Go Test

In the go/no go test (Table 6b), the main effect of group was not significant for RTs, indicating that a similar amount of time was needed to respond to selected stimuli; however, the SDs of RTs were significantly higher for the OSA patients, showing increased instability of performance (86.8 vs. 72.9 ms, F_(1,64)_ = 8.45*, *p* < 0.01). Moreover, the OSA patients tended to produce fewer valid responses (21.3 vs. 23.7, F_(1,64)_= 3.89, *p* = 0.05), made significantly more omissions than controls (0.28 vs. 0.03, F_(1,64)_ = 5.84*, *p* = 0.01) and tended to have more false reactions (0.94 vs. 0.44, F_(1,64)_ = 3.5, *p* = 0.06); these data indicate an alteration of their selective attention process.

When analyses were replicated with the cognitive reserve index as covariate, all results were replicated, and the covariate was not significant (F < 1).

Finally, in repeated measures ANOVA with three subgroups of patients (AHI^++^D^+^, AHI^++^D^−^, and AHI^+^D) no main effects or interactions reached significance.

### 3.5. Divided Attention

In the divided attention test (Table 6c), the main effect of group was not significant for RTs but was significant for SDs of RTs, with the OSA patients presenting significantly greater instability in attention performance than controls (268.27 vs. 214.50 ms, F_(1,64)_ = 15.32*, *p* < 0.001). The OSA patients tended to make fewer valid responses (28.1 vs. 29.5, F_(1,64)_ = 3.51, *p* = 0.06). Concerning errors, patients made significantly more false responses than the controls (3.1 vs. 1.4, F_(1,64)_ = 5.61*, *p* < 0.05), indicating impaired selectivity or reduced control in responding. The number of omissions tended to be higher for the OSA patients than those of the controls (2.75 vs. 1.8, F_(1,64)_ = 3.51, *p* = 0.06), but the two groups were comparable in the number of delayed responses.

**Figure 6:**
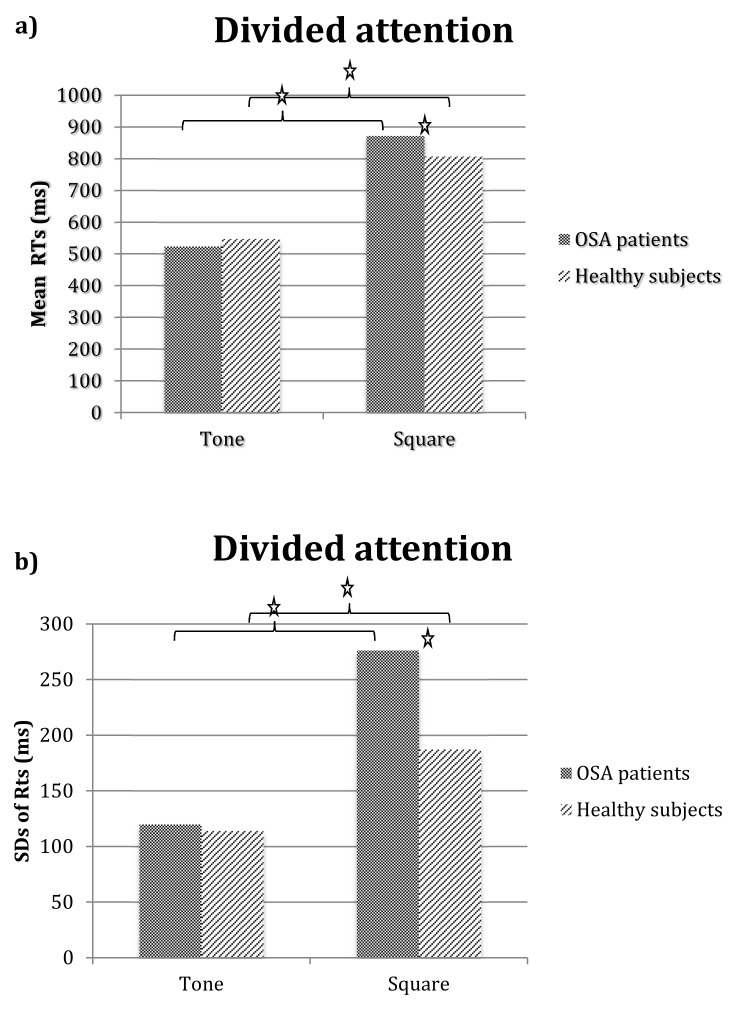
Mean reaction times (**panel a**) and standard deviations of reaction times (**panel b**) of OSA patients and controls in auditory and visual tasks of divided attention test. Legend: Stars indicate significant differences.

Figure 6 show RTs (panel a) and SDs of RTs (panel b) obtained for the OSA patients and the controls in the auditory and visual tasks of the divided attention test. Repeated measures ANOVA with group as the between factor and task (auditory vs. visual) as the within factor showed a main effect of task on RTs (F_(1,64)_ = 252.14*, *p* < 0.0001), indicating longer RTs for the visual than the auditory task; the group-by-task interaction was also significant (F_(1,64)_ = 5.49*, *p* < 0.05). Exploration of means showed that in both groups, RTs were higher on the visual than the auditory task (at least *p* < 0.0001); however, the OSA patients had significantly higher RTs on the visual task than the controls (*p* < 0.01) and their RTs on the auditory task did not differ from those of the controls. The analysis of SDs showed a similar effect: there was a main effect of group (F_(1,64)_ = 16.11*, *p* < 0.001), with the OSA patients obtaining higher values than the healthy controls (198.2 vs. 151.02, respectively), task (F_(1,64)_ = 100.69*, *p* < 0.0001), and higher values on the visual than the auditory task (232 vs. 117, respectively) and a significant group-by-task interaction (F_(1,64)_ = 13.52*, *p* < 0.001). Exploration of means showed that, in both groups, SDs were higher on the visual than the auditory task (at least *p* < 0.001); however, the OSA patients’ SDs values on the visual task were higher than all others (at least *p* < 0.0001), indicating their greater state of instability or lapses of attention during the visual task.

The analysis of valid responses in the auditory and visual tasks showed a tendency toward significance of the main effect of group (F_(1,64)_ = 3.11, *p* = 0.08), but not of task or group-by-task interactions (F < 1), indicating a comparable number of valid responses across the visual and auditory tasks for both groups. Concerning errors, omissions tended to be higher for the OSA patients (1.35 vs. 0.88, F_(1,64)_ = 3.44, *p* = 0.06) and were significantly higher on the visual than the auditory task (1.72 vs. 0.52, respectively, F_(1,64)_ = 25.91*, *p* < 0.0001); however, the group-by-task interaction was not significant (F < 1).

False reactions and delayed responses showed no main effect of group, task, or group-by-task interactions (all F < 1).

When analyses were replicated with the cognitive reserve index as covariate, all results were replicated, and the covariate was not significant (F < 1).

Finally, the repeated measures ANOVA with three sub-groups of patients showed only a main effect of task for RTs, SDs and omissions, indicating higher values in the visual than the auditory task for RTs (889.56 vs. 530.59, respectively; F_(1,28)_ = 136.24*, *p* < 0.0001), SDs of RTs (281.36 vs. 122.53, respectively; F_(1,28)_ = 78.90*, *p* < 0.0001), and omissions (2.16 vs. 0.78, respectively; F_(1,28)_ = 12.48*, *p* < 0.001). No effects or interactions reached significance in the analysis of delayed responses (all F < 1), while false reactions were absent.

### 3.6. Correlations between Clinical Variables and Attention Parameters

Table 7 shows only the significant correlations between AHI, hypoxemia parameters, ESS scores, and speed performance (RTs and SD of RTs) and accuracy (total number of errors) for the different TAP attention tests. The pattern of results is scattered, but it seems that both sleepiness and some variables of hypoxemia correlated significantly with RTs of tasks requiring intensive attention processes, such as alertness mean RTs (or the index for phasic alertness reporting increased RT to warning) and vigilance mean RTs for the different intervals. ESS, T90, ODI, and AHI values correlated positively with RTs, indicating that higher scores were associated with longer response times. The mean SaO_2_ correlated negatively, indicating that lower values were associated with longer RTs. The ESS scores also significantly correlated with RTs of the selective attention task but not with those recorded in the divided one. Regarding RT variability, only ESS scores were associated with SDs of RTs of both alertness and vigilance, indicating a link between instability of performance and sleepiness. The T90 and NSaO_2_ values correlated with SDs of the go/no go tests. Regarding errors, AHI, hypoxemia parameters, and ESS scores correlated with errors committed at longer intervals of the vigilance test, but only hypoxemia parameters correlated with errors committed in the divided attention test (the visual component). The direction of correlations is the one expected.

## 4. Discussion

Although attention deficits are frequently reported in OSA patients, results are inconclusive and affected by the heterogeneity of sampling and methodology. In the present study, we updated the attention profiles of patients with moderate to severe OSA using an extensive computerized battery that assessed both intensive and selective aspects of attention in speed and accuracy parameters.

The results suggest that a spectrum of attention processes are altered in this population, at least in patients with moderate to severe OSA. In the alertness test, the OSA patients presented higher RTs and a greater instability of the level of performance as compared with the healthy controls. The vigilance data revealed an impairment of attention maintenance after 15 min of testing, with normal timely responses but a significant decrease in the number of valid responses and increased omissions in the 20 to 30 min intervals. In addition, selective aspects of attention were defective. In the go/no go tests, although OSA patients needed a similar amount of time to respond, they showed instability in RTs and significantly more omissions and false responses than the controls, showing deficits in enhancing the processing of attended stimuli and inhibiting irrelevant ones. Difficulties were also present in coping with dual tasks; OSA patients showed a greater instability of response times and lower accuracy due to errors, mainly omissions. Interestingly, our data showed that there was a greater decrement in visual than auditory performance when OSA patients were engaged in dual tasks.

Thus, it seems that OSA patients present deficits in remaining awake in monotonous situations, and also their ability to remain attentive in more demanding conditions is impaired. The picture of results was stable and did not vary as a function of patients’ cognitive reserve. Indeed, it seems that although cognitive reserve can hypothetically cope with neurocognitive deficits related to OSA, patients can still exhibit attentional impairment. Moreover, RT is not the best index for revealing attention impairments; in fact, error analysis and performance stability need to be considered.

Speculating on possible components and processes underlying attention dysfunction in OSA patients, some authors have suggested that RT lengthening and variability are expressions of significant slowing down in the motor component of RTs (i.e., selection of the appropriate motor response, [24]) or primary arousal problems [26] rather than impairments in decision processes or focused attention. Actually, we also found longer alertness RTs. Slower reactions can be a significant handicap in daily life. However, it can reflect either a general reaction speed attenuation or difficulty in maintaining high response readiness (intrinsic alertness) in a specific test. This seems to be the case, i.e., in the other attention subdomains, RTs were not selectively compromised in our sample, while instability of performance, increased omissions and false responses seemed to characterize OSA performance changes. Of course, other studies are necessary to assess whether omissions and false reactions arise from motor slowing [24], but the data do not seem to be clearly explicable by this component. Instability to perform, instead, can be compatible with impaired arousal. Following the “state instability” hypothesis, increased variability can be due to the influence of sleep-initiating mechanisms on the endogenous capacity to maintain attention and alertness, thereby creating an unstable state that fluctuates within seconds, with neurobehavioral consequences. In other neurological disorders [81], RT variability was found to be linked with EEG and behavioral markers of cognitive fluctuations (e.g., falls, falling asleep, and disorganized thinking). Regarding omissions and false responses, unlike RTs, it seems that they captured performance changes in the go/no go and divided attention tasks. Both tasks are complex, i.e., the go/no go task requires control of attention focus, inhibiting distractibility, and the divided attention task implies sharing/switching available resources between competing tasks. Focused attention and inhibitory control are related to prefrontal functions (e.g., [82,83]). In addition, divided attention entails the activation of a complex network including dorso- and ventrolateral prefrontal structures, superior and inferior parietal cortex, and anterior cingulated gyrus [84]. OSA patients have shown decreased activation in brain regions involved in the go/no go task [85]. Moreover, dysfunction of prefrontal regions of the brain cortex has largely been postulated in OSA patients (e.g., [12,58] In a recent study by our group on attention deficits in neurological patients, those suffering from partial anterior circulation infarcts were found to be more impaired in all attention tasks [86]. Finally, it is worth noting that the cognitive control of wakefulness and arousal (i.e., intrinsic alertness) arises from a network that includes cortical and subcortical structures (dorsolateral prefrontal cortex, anterior cingulate gyrus, parietal cortex, thalamus, and brainstem) [72,73,75]. Overall, the present data are coherent with the idea that attention is a complex system of specific abilities highly susceptible to different kinds of damage related to both bottom-up and top-down neural mechanisms.

Overall, the present data confirm a broad range of shaded attention difficulties and suggest that conflicting results, clearly reported in the literature (see [9,14,32]), could depend on sampling differences between studies (e.g., age, OSA severity, and presence of comorbidities, for the role of comorbidities see [19] but also other factors. One factor is certainly the greater variability present in the data, which has usually been ignored and could result in different average performance (making average RTs unreliable) from one study to another (see, e.g., [87,88], in a different domain). In the present study, we also investigated the instability of attention performance taking into account performance variability (through the evaluation of subjects’ SDs of RTs), and we found attention instability among the OSA patients. Moreover, the data represent important interindividual variability. In the present study, we adopted strict criteria for inclusion and exclusion and enrolled people under 65 years of age without relevant comorbidities, general neurocognitive impairment, and with moderate to severe OSA (mild OSA was excluded) and found a broad range of attention difficulties more clearly characterizing a subgroup of patients.

In fact, although a strong correlation was found between AHI and the parameters of nocturnal hypoxemia, patients with severe AHI could differ also in the degree of nocturnal hypoxemia. Patients with severe OSA and greater nocturnal hypoxemia presented a more compromised attention profile, at least in the intensive processes, with longer RTs in the alertness and vigilance tasks and a higher rate of omissions in medium and long intervals in the vigilance test, although they did not differ in subjective sleepiness. Furthermore, parameters of oxygenation correlate with RTs and error rates in the alertness and vigilance tests and the divided attention test. Overall it seems that parameters of oxygenation describe a highly compromised phenotype of OSA (see also [60]) and have a significant role in determining sustained attention deficits (see also [36,59]). As far as sleepiness is concerned, since we did not perform polysomnography to assess sleep architecture and quality, we were able to capture only subjective sleepiness due either to sleep loss and sleep fragmentation, or to hypoxic damage to sleep-wake brain regions induced by CIH. However, it is worth noting that our data fail to show any significant correlation between nocturnal parameters of oxygenation and ESS, or any significant differences in sleepiness among the three OSA phenotypes. Although it is possible that laboratory measures used to evaluate the severity of OSA and hypoxemia could have been ineffective in capturing subjective sleepiness, we observed that ESS scores, although they did not correlate with oxygenation parameters, significantly correlated with alertness and vigilance and go/no go RTs (but not with divided attention) and with errors in the vigilance task. The fact that only the ESS scores correlated with SDs of the intensive attention test, suggests that sleepiness can be related to attention instability, as measured by RT variability. Instability due to sleep loss was already described (e.g., [89]). Of course, we must still explain why ESS did not correlate with divided attention performance. In any case, our data indicate that patients’ subjective level of EDS underpins some aspects of attention functioning, and sleepiness places an additional load on attention processing.

Overall, the results reflect the multifaceted mechanisms of attention dysfunction in OSA and the data corroborate the idea that both EDS and CIH contribute to the impairment of attention processes [47].

However, a note of caution is needed with regard to generalizing the present results, due to some study limitations. The sample size was not large enough and was not representative of women, therefore, the data need to be confirmed by a large-scale study. Moreover, we did not perform a sleep study to assess the quality and architecture of sleep, so our measure of sleepiness could have been related both to sleep fragmentation or loss and to other factors. Finally, the study was not corroborated by a neuroimaging study, which would better disclose the dysfunctional neural mechanisms. At the same time, it would be interesting, in a future study, to examine other cognitive functions, such as memory and executive functioning, among an OSA population.

## 5. Conclusions and Future Perspectives

In conclusion, we hope that this study improves the understanding of attention dysfunctions in OSA patients. Our results support the finding that a broad range of attention deficits, in addition to vigilance, are compromised in OSA patients (at least in severe cases of OSA). The impacts of the different attention deficits are clear, i.e., reduced response readiness in situations of alertness, decreased long-lasting attentiveness, loss of control over the attention focus, and difficulties in dividing attention (divided attention situations are the rule rather than the exception in everyday life), resulting in a serious handicap in daily and working life. For many patients, the ability to work is limited or fully lost, specifically as a result of their impaired attention performance. Therefore, a differential diagnosis of attention is of particular importance, and we hope that our data could affect clinical and medical-legal practices. For example, in the context of clinical, medical-legal, and occupational practices, our data indicate that in order to better evaluate daytime attention impairment (potentially associated with work and driving accidents, but also relevant for clinical decision making), it is important to use longer vigilance tasks, assess several attention skills (not only intensive dimensions), use accuracy and stability measures to capture performance changes, and consider more than the usual indices of severity (e.g., AHI) in predicting daytime consequences for OSA patients.

## 6. Highlights

- OSA patients suffer from both intensive and selective attention deficits;
- Reaction time (RT) alone is ineffective for tracking attention failures of OSA patients;
- Error analysis and stability of performance capture attention impairments of OSA patients;
- Longer vigilance tasks are needed to detect vigilance impairments of OSA patients;
- OSA patients fail not only to remain awake in monotonous situations, but also to be attentive in more demanding situations.

## Figures and Tables

**Table 1:** Demographic and clinical data of obstructive sleep apnea (OSA) patients and healthy controls.

	OSA Patients (*n* = 32)		Healthy Controls (*n* = 34)			
	Mean	Range	Mean	Range	F_(1.64)_	*P*
**Age**	53.2 ± 9.7	32–65	50.3 ± 6.14	39–62	2.1	n.s.
**Education**	10.4 ± 3.4	5–17	11.7 ± 2.7	8–18	3.0	n.s.
**Sex (M/F)**	26/6		30/4			
**MMSE**	28.2 ± 1.3	23–31	28.4 ± 0.9	27–30	0.57	n.s.
**Cognitive reserve**	102.12 ± 15.91	82–149	103.9 ± 10.2	64–126	0.31	n.s.

**Table 2:** Polygraphic recording indices.

	Mean	Range
AHI (events/h)	45.3 ± 22.9	16.1–69.6
Mean SaO_2_ (%)	91.7 ± 4.18	78–96
ODI (events/h)	49.78 ± 24.75	5–93
T_90_ (%)	27.34 ± 28.82	0.1–94
Nadir SaO_2_ (%)	68.5 ± 16.0	18–89

**Table 3:** Pearson correlations between polygraphic recording indices and Epworth Sleepiness Scale (ESS) in entire sample of OSA patients.

	AHI	MeanSaO_2_	ODI	T_90_	NadirSaO_2_
ESS	0.066	−0.212	0.049	0.208	−0.143
AHI		−0.698 **	0.888 **	0.769 **	−0.492 **
MeanSaO_2_	−0.670 **		−0.680 **	−0.918 **	0.611 **
ODI	0.888 **	−0.680 **		0.797 **	−0.587 **
T_90_	0.769 **	−0.918 **	0.797 **		−0.742 **
NadirSaO_2_	−0.492 **	0.611 **	−0.587 **	−0.742 **	

** *p* < 0.001.

**Table 4:** Classification of OSA patients as a function of apnea-hypopnea index (AHI) and desaturation severity. AHI^++^, severe OSA and AHI^+^, moderate OSA.

	AHI^++^	AHI^+^
Desaturators (D^+^)	10	1
Nondesaturatos (D^−^)	13	8

**Table 5:** Mean (and standard deviation) of demographic and clinical data for three groups of OSA patients.

	AHI^++^D^+^(N = 10)	AHI^++^ D^−^ (N = 13)	AHI^+^ D^−^ (N = 8)	F_(2,28)_	*P*
Age	56.5 ± 9.9	52.0 ± 8.9	49.7 ± 10.7	1.16	n.s
Education	9.2 ± 2.8	12.5 ± 3.5	8.9 ± 2.7	1.20	n.s
MMSE	28.3 ± 1.4	28.8 ± 0. 70	27.43 ± 1.9	2.61	n.s
Cognitive reserve	97.2 ± 9.2	107.0 ± 15.7	100.3 ± 23.4	1.11	n.s
BMI	36.3 ± 4.2	32.3 ± 4.6	36.1 ± 14.1	0.83	n.s
ESS	13.5 ± 2.9	12.4 ± 3.7	12.1 ± 1.6	0.55	n.s
AHI (events/h)	64.3 ± 19.7	47.8 ± 12.5	21.1 ± 4.7	20.8	0.0001
Mean SaO_2_ (%)	89.9 ± 4.4	91.9 ± 4.9	94.4 ± 1.7	7.56	0.01
ODI (events/h)	74.91 ± 13.2	49.01 ± 14.1	19.03 ± 11.7	39.44	0.0001
T_90_ (%)	58.9 ± 19.5	16.3 ± 21.1	4.4 ± 4.3	24.98	0.0001
NadirSaO_2_ (%)	56.9 ± 16.1	71.1 ± 13.2	78.7 ± 11.9	5.87	0.01

Legend: n.s = not significant; the value after ± indicate the standard deviation.

**Table 6:** Performance of OSA patients and healthy controls on the Test for Attention Performance subtests.

	OSA Patients (n = 32)	95%CI	Healthy Controls (n = 34)	95%CI	F_(1,64)_	*P*
	Mean ± SD	95% Confidence Interval	Mean ± SD	95% Confidence Interval		
(a) Vigilance test (0–30 min)						
Median RT, ms	454.5 ± 114.8	411.4–497.6	460.1 ± 99.0	428.3–491.9	0.04	n.s.
SD of the RT, ms	111.0 ± 37.33	97.5–124.5	108.0 ± 35.34	96.3–120.9	0.7	n.s.
Valid responses	31.6 ± 5.30	29.7–33.5	33.8 ± 2.49	32.8–34.6	4.5 *	0.05
Omissions	3.62 ± 4.6	1.9–5.2	1.2 ± 2.2	0.4–2.0	7.3 *	0.01
False reactions	3.25 ± 4.66	1.5–4.9	2.03 ± 5.48	0.1–3.9	0.9	n.s.
(b) Go/No Go test						
Median RT, ms	508.5 ± 78.5	479.5–537.4	509.7 ± 62.4	490.1–529.4	0.01	n.s.
SD of the RT, ms	86.8 ± 21.3	79.2–94.5	72.9 ± 17.5	66.8–79.0	8.5 *	0.01
Valid responses	21.3 ± 0.9	20.4–23.6	23.7 ± 0.49	23.4–23.8	3.9	0.05
Omissions	0.28 ± 0.6	0.1–0.4	0.03 ± 0.17	0.03–0.08	5.85 *	0.05
False reactions	0.94 ± 1.4	0.4–1.4	0.44 ± 0.7	0.2–0.7	3.5	0.06
(c) Divided test						
Median RT, ms	645.9 ± 100.9	609.5–682.3	440.3 ± 70.6	615.6–664.9	0.1	n.s.
SD of the RT, ms	268.3 ± 64.8	244.8–291.6	214.5 ± 44.2	198.8–230.2	15.3 *	0.001
Valid responses	28.1 ± 3.43	26.8–29.2	29.5 ± 2.79	28.5–30.4	3.5	0.06
False reactions	3.1 ± 3.98	1.6–4.5	1.4 ± 1.35	0.9–1.8	5.6 *	0.05
Omissions	2.75 ± 2.05	2.0–3.4	1.8 ± 2.15	1.0–2.5	3.6	0.06
Delayed reactions	0.97 ± 0.52	0.7–1.1	0.75 ± 0.56	0.5–0.9	2.7	n.s.

Legend: n.s. = not significant; the value after ± indicate the standard deviation. * indicates F values exceeding the critical value according to post-hoc power analysis, given the probability of a Type I error set at 0.05 and a Type II error set at 0.80.

**Table 7:** Significant correlations (Pearson) between apnea-hypopnea index, oxygenation parameters, Epworth Sleepiness Scale, and Test for Attention Performance scores for the OSA patients.

	AHI	MeanSaO_2_	ODI	T_90_	NSaO_2_	ESS
Alertness RT						0.373 *
Alertness (no W) RT						0.387 *
Index Phasic Alertness	0.505 **	−0.433 *	0.480 **	0.469 **		
Vigilance 1–5 RT						0.377 *
Vigilance 5–10 RT						0.450 *
Vigilance 10–15 RT		−0.436 *		0.434 *		
Vigilance 15–20 RT						0.377 *
Vigilance 20–25 RT				0.409 *		
Vigilance 25–30 RT						0.368 *
Go-No Go RT						0.376 *
Alertness SD						0.434 *
Alertness (no W) SD						0.413 *
Alertness (W) SD						0.414 *
Vigilance 5-10 SD						0.431 *
Go-No Go SD				0.378 *	−0.365 *	
Vigilance 1–5 Err						0.384 *
Vigilance 10–15 Err			0.379 *			
Vigilance 15–20 Err			.			
Vigilance 20–25 Err	0.374 *		0.515 **	0.452 *	−0.424 *	0.436 *
DividedSQ Err		−0.415 *	0.375 *	0.421 *	−0.373 *	

Legend: Alertness (W) = alertness with warning; Alertness (no W) = alertness no warning; DividedSQ = divided attention, squares condition (i.e., visual task). ** *p* < 0.01 and * *p* < 0.05.
